# MOBILITY DEVICE USE MODERATED BIDIRECTIONAL ASSOCIATION OF FEAR OF FALLING WITH HOMEBOUND STATUS

**DOI:** 10.1093/geroni/igae098.0234

**Published:** 2024-12-31

**Authors:** Qianyuan Li, Wenting Peng, Shuomin Wang, Huili Yang, Haihong Zhang, Minhui Liu

**Affiliations:** Central South University, Changsha, Hunan, China (People’s Republic); University of Washington, Seattle, Washington, United States; Central South University, Changsha, Hunan, China (People’s Republic); Ningxia Medical University, Yinchuan, Ningxia, China (People’s Republic); Ningxia Medical University, Yinchuan, Ningxia, China (People’s Republic); Ningxia Medical University, Yinchuan, Ningxia, China

## Abstract

Previous studies showed fear of falling (FOF) is associated with homebound status, given that they have shared risk factors such as falls. However, the bidirectional relationship between fear of falling and homebound status and possible moderators remain unknown. Using the Year 2015-2019 waves of the National Health and Aging Trends Study, our sample included 3,983 community-dwelling older adults who had completed data on FOF and homebound status. We aimed to examine 1) whether FOF was associated with homebound status bidirectionally and 2) whether mobility device use moderated this bidirectional association. FOF was categorized as no FOF, FOF without fear-related activity restriction (FAR) and FAR. Based on the frequency, difficulty, and independence of outdoor mobility, homebound status was classified as non-homebound, semi-homebound, and homebound. The lagged generalized estimation equation models revealed that FOF without FAR (OR=1.34, 95% CI=1.19-1.52) and FAR (OR=1.62, 95% CI=1.38-1.91) increased the risks of semi-homebound/homebound status. Mobility device use moderated the longitudinal effects of FAR on homebound status with a statistical significance. Additionally, semi-homebound predicted the risks of FOF (OR=1.36, 95% CI=1.21-1.53), and its association was moderated by mobility device use. Overall, FOF is a risk factor for homebound status, and vice versa, and the risks may differ by the use of mobility devices. To prevent the progression of homebound status or FOF, future targeted strategies or community-based interventions should consider the fear of falling and homebound status simultaneously and identify the high risks of older adults who do not use mobility devices.

